# KeyPathwayMiner 4.0: condition-specific pathway analysis by combining multiple omics studies and networks with Cytoscape

**DOI:** 10.1186/s12918-014-0099-x

**Published:** 2014-08-19

**Authors:** Nicolas Alcaraz, Josch Pauling, Richa Batra, Eudes Barbosa, Alexander Junge, Anne GL Christensen, Vasco Azevedo, Henrik J Ditzel, Jan Baumbach

**Affiliations:** 1 Max Planck Institute for Informatics, Saarbrücken, Germany; 2 Cluster of Excellence for Multimodal Computing and Interaction, Saarland University, Saarbrücken, Germany; 3 Department of Biochemistry and Molecular Biology, University of Southern Denmark, Odense, Denmark; 4 Department of Mathematics and Computer Science, University of Southern Denmark, Odense, Denmark; 5 Department of Cancer and Inflammation Research, Institute of Molecular Medicine, University of Southern Denmark, Odense, Denmark; 6 Department of Oncology, Odense University Hospital, Odense, Denmark; 7 Institute of Biological Sciences, Laboratory of Molecular and Cellular Genetic, Federal University of Minas Gerais, Belo Horizonte, Brazil; 8 Center for non-coding RNA in Technology and Health, Section for Animal Genetics, Bioinformatics and Breeding, University of Copenhagen, Frederiksberg, Denmark

**Keywords:** Network enrichment, Protein-protein interaction, Multi-omics, Key pathways

## Abstract

**Background:**

Over the last decade network enrichment analysis has become popular in computational systems biology to elucidate aberrant network modules. Traditionally, these approaches focus on combining gene expression data with protein-protein interaction (PPI) networks. Nowadays, the so-called omics technologies allow for inclusion of many more data sets, e.g. protein phosphorylation or epigenetic modifications. This creates a need for analysis methods that can combine these various sources of data to obtain a systems-level view on aberrant biological networks.

**Results:**

We present a new release of KeyPathwayMiner (version 4.0) that is not limited to analyses of single omics data sets, e.g. gene expression, but is able to directly combine several different omics data types. Version 4.0 can further integrate existing knowledge by adding a search bias towards sub-networks that contain (avoid) genes provided in a positive (negative) list. Finally the new release now also provides a set of novel visualization features and has been implemented as an app for the standard bioinformatics network analysis tool: Cytoscape.

**Conclusion:**

With KeyPathwayMiner 4.0, we publish a Cytoscape app for multi-omics based sub-network extraction. It is available in Cytoscape’s app store http://apps.cytoscape.org/apps/keypathwayminer or via http://keypathwayminer.mpi-inf.mpg.de.

## Background

The massive increase in high-quality and publicly available biological databases over the last decade has led to network enrichment analysis becoming a comprehensively used approach to study entire lists of proteins and the effect of their interactions instead of single functional analyses. The goal is to identify differentially expressed sub-networks in an organism’s interaction network. These sub-networks are commonly referred to as active modules [1],[2] and key pathways [3],[4], for instance.

Most *de novo* network enrichment methods have traditionally been focusing on combining gene expression microarray data with PPI networks. Advancements in high throughput techniques have made it possible to produce various omics datasets in addition to expression data, for example DNA-methylation and other epigenetic modifications, single nucleotide polymorphisms (SNPs), gene copy number variations (CNVs), or protein expression and post-translational modifications, amongst others. We seek to integrate all of the available omics data measured in a certain experiment to account for the entangled nature of biological process regulation. Recently, some attempts have been made to combine multiple omics datasets to provide an integrative enrichment platform, e.g. [5],[6]. However, there is no app available for the combined analysis of multiple omics data sets together with biological networks in Cytoscape, which is the standard tool for bioinformatics systems biology network analysis [7].

Previously, we have developed KeyPathwayMiner, a method to identify aberrant sub-networks based on combining a single omics study and a PPI network in Cytoscape release series 2. This was accomplished by a selection of algorithms to solve a graph-based combinatorial optimization problem: Extraction of a maximal connected sub-network where all nodes but at most k are differentially expressed (deregulated) in all patients/cases but at most l [4]. KeyPathwayMiner comes with a set of heuristics (ant colony optimization), as well as exact (fixed-parameter) algorithms that combine a statistical evaluation and discretization on the omics data and PPI network. The KeyPathwayMiner strategy was explained and evaluated in detail in [4],[8],[9].

**Figure 1:**
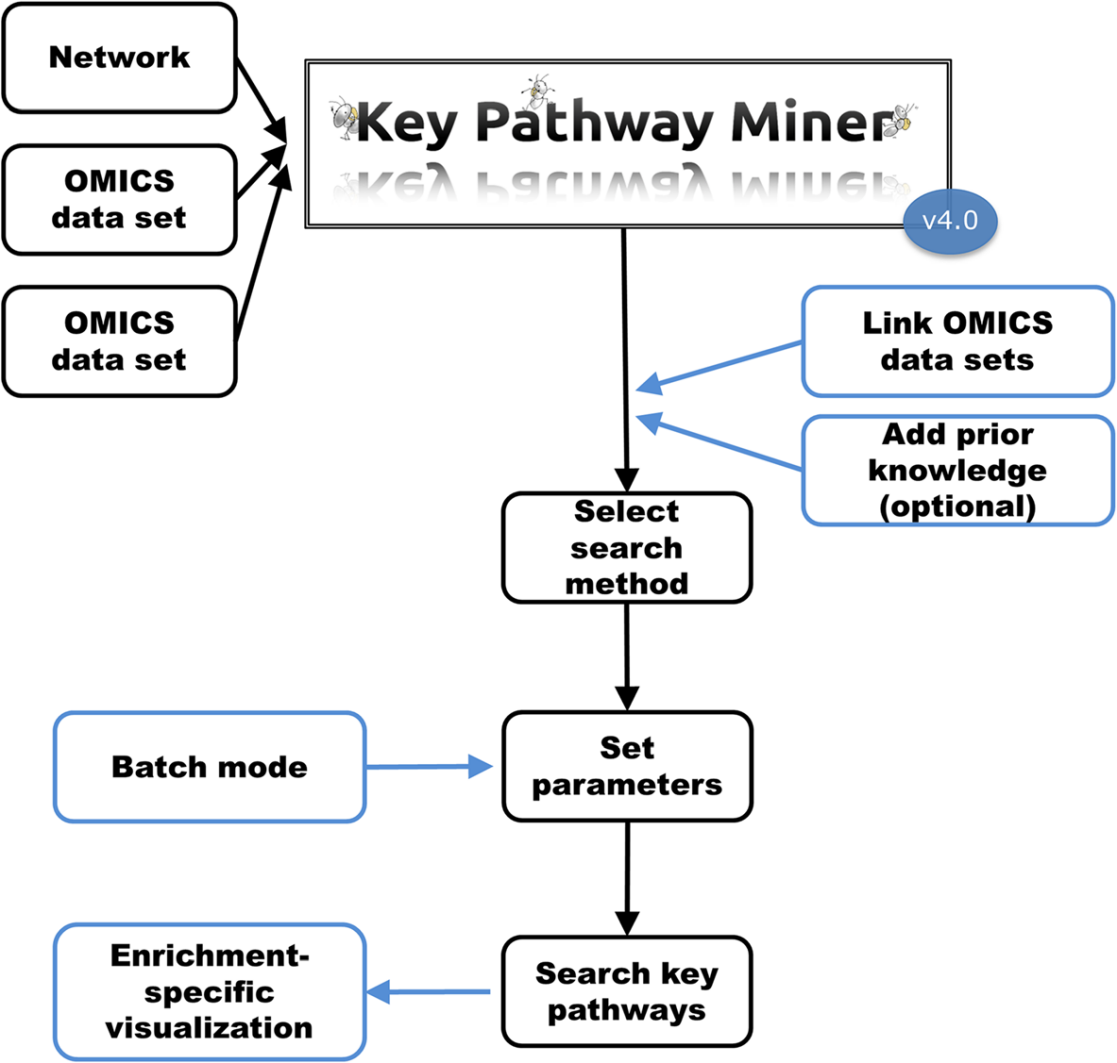
**KeyPathwayMiner workflow.** The previous versions of KeyPathwayMiner allowed network enrichment with a single dataset. KeyPathwayMiner 4.0 can integrate multiple datasets and provides advanced visualization. The new features of KeyPathwayMiner 4.0 are highlighted (blue borders).

Here, we account for compatibility with the new Cytoscape release 3 [10], which required redesign of the previous app code to comply with the new API library. With the new KeyPathwayMiner 4.0 app, we also implemented a set of extensions to the previous version, which broaden the applicability of KeyPathwayMiner and increases its usability. The usual workflow through KeyPathwayMiner is depicted in Figure 1. New features are highlighted in blue color. We added functionality to (1) provide multi-dataset support, (2) integrate existing knowledge, and (3) enhance visualization and ease of parameter setting. As an application case, we describe a colorectal cancer case study that makes use of the multi-omics feature by integrating gene expression and DNA methylation data. We also provide step-by-step instructions on how to use KeyPathwayMiner 4.0 in Additional file 1.

## Implementation

### Combining multiple omics data sets

We assume discretized omics data sets, i.e. they have been statistically transformed into indicator matrices. An indicator matrix describes if a specific gene/protein is significantly altered in its expression for a specific case compared to a set of controls. This step largely depends on the study design and the applied omics technologies. It has to be performed for each data type individually.

While integrating the different omics datasets the number of cases/samples in each of the studies has to be accounted for. If all omics studies come from the same cases and, thus, have the same number of cases, one indicator matrix can be generated for each study. Afterwards, all these matrices are combined into a single one by a set of particular criteria. Consider a gene *P*, which is active in case *Q1* (expression matrix *M1)*, and the same gene is not active in case *Q2* (expression matrix *M2)*. One may apply a logical “OR”, for instance, such that P counts as active in the combined matrix *Mc = M1 OR M2*.

However, for unconnected datasets, where the number of cases doesn’t match (e.g. different sample sets used for expression data and methylation data), KeyPathwayMiner was extended to accept multiple matrices. The user now can specify the maximum number of non-active cases (*L* exceptions) for each matrix in order for each gene to be considered active. For simplicity, let’s assume we have two matrices *M1* and *M2* with differing number of cases. Then parameters *L1* for *M1* and *L2* for matrix *M2* can be specified. After selecting the desired logical connector, for example the logical “AND”, then a gene will be considered active if it’s corresponding entry in *M1* contains at most *L1* non-active cases AND it’s corresponding entry in *M2* contains at most *L2* non-active cases. Finally, KeyPathwayMiner 4.0 will then scan for maximal connected sub-graphs where all nodes but at most *K* are active in all but *L1* cases in *M1* and *L2* cases in *M2*.

**Figure 2:**
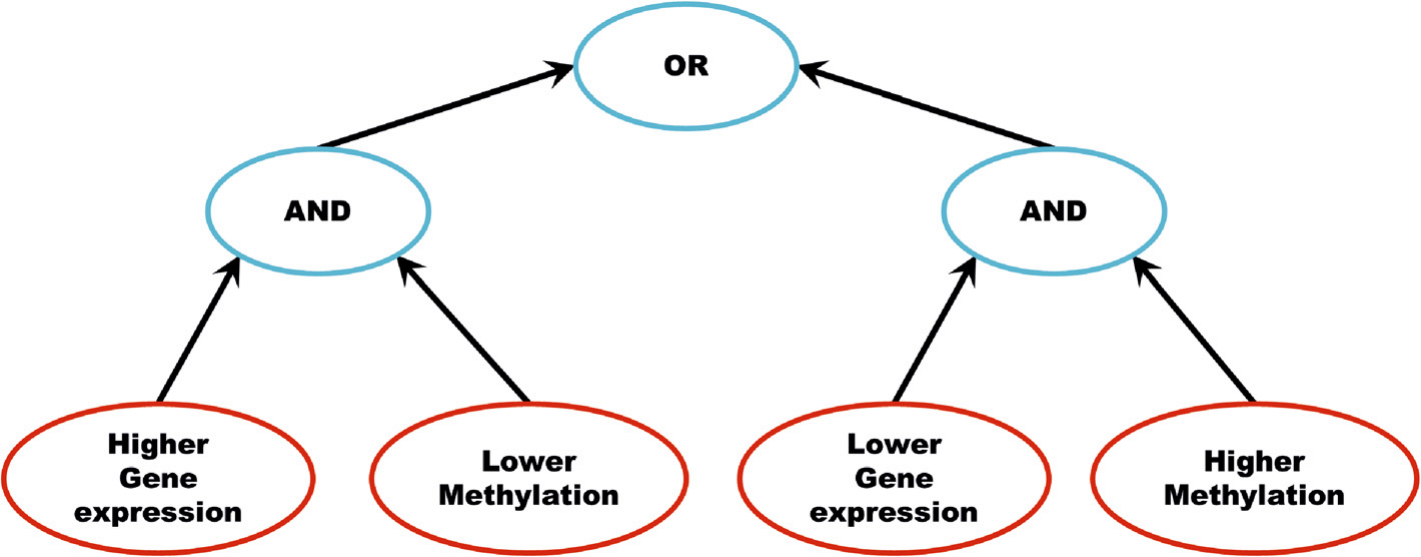
**Combined formula for the colorectal cancer case study.** We combined the four matrices such that the key pathways represent aberrant gene expression as well as irregular DNA methylation.

The KeyPathwayMiner 4.0 app now offers a graphical user interface to apply logical connectors (AND, OR, XOR) to merge the data sets (see Figure 2 for an example, as applied on the colorectal cancer application case), to project them on the network, and to perform the enrichment.

### Integration of prior knowledge

We now offer text fields to input two lists of node IDs, a positive and a negative one. The nodes in the positive list will be treated as non-exception nodes by the algorithms and will bypass the parameter constraints. Therefore, the nodes specified in the positive list are more likely to pop up in a solution, i.e. the list of deregulated sub-networks (key pathways). Note that positive lists may generally lead to larger key pathways. Inversely, the nodes from the negative list will always be treated as exception nodes and are less likely to be present in a solution. Thus, these lists will bias the search algorithms to prefer or avoid a specified set of genes/proteins (i.e. nodes). This feature can be used to reflect existing background knowledge. Genes or proteins that are known to be unrelated may be avoided. Solutions containing nodes known to play a role in a certain disease, for instance, may be favored.

### Enrichment-specific visualization

Previous software versions presented the results as a list of deregulated key pathways for one set of parameters. The new app comes with two improvements. First, it can pre-compute solutions for systematically varying combinations of the main parameters *k* and *l*. Second, to assist in parameter optimization, we implemented a node-coloring scheme such that the color intensity of a node varies with regard to the number and size of key pathways containing it. After the pathway search is complete, the user can see darker and lighter blue areas in the Cytoscape visualization of the network. By clicking on one combination, the user can visually inspect the effect of changing parameters on the size (and location) of the reported sub-networks (the higher *k* and *l*, the bigger the blue areas in the network). The node coloring, thus, visually supports the fine-tuning of optimal parameter combinations. Several other visualization schemes are implemented in the app (not discussed here).

### Case study

In the following sections, we describe a case study, to introduce the utility of KeyPathwayMiner 4.0 and to explain how the Cytoscape app can be used to analyze multiple omics datasets conjointly and together with a network given and displayed with Cytoscape release 3. Additional file 1 provides a step-by-step description on how to install Cytoscape, KeyPathwayMiner, and how to analyze the dataset (a sample file and application case is provided with the additional material). For further information, please refer to the web site (http://keypathwayminer.mpi-inf.mpg.de) and click at “App for Cytoscape 3” to receive a detailed description of all features. This page will be kept up-to-date and describe future software updates and improvements.

### Background

In this case study, we used the new KeyPathwayMiner 4.0 app to perform a multi-omics analysis of colorectal cancer. Colorectal cancer accounts for high cancer mortality and morbidity [11]. We used gene expression and DNA methylation data available in public domain to study the epigenetic regulation of gene expression in cancer patients. Epigenetic regulations plays a significant role in controlling gene expression in cancerous cells specifically in colorectal cancer [12].

### Data & data processing

#### The protein-protein interaction network

The protein-protein interaction network was obtained from HPRD [13]. The network consisted of 9,427 nodes and 36,812 edges (Additional file 2).

#### OMICs data sets

We downloaded the gene expression data and DNA methylation data from The Cancer Genome Atlas (TCGA) [11]. We used the preprocessed data available at the TCGA data portal: 155 samples and 19 normal samples as gene expression data as well as 291 samples and 38 normal samples as DNA methylation dataset. For both these data sets, indicator matrices were computed by comparing their expression values to the normal samples. We generated four indicator matrices representing up/down gene expression and high/low DNA methylation for each gene in each sample (Additional file 3) and combined them as depicted in Figure 2.

## Results

The objective was to identify the signaling component that is active in most of the samples and has aberrant DNA methylation as well as aberrant gene expression. Since DNA-methylation impairs a gene’s transcriptional potential we connected the four matrices in such a way that the up-regulation of gene expression is associated with hypo-methylation and down-regulation of gene expression is associated with hyper methylation, as shown in Figure 2.

With this combined formula, we computed the pathways using the HPRD protein-protein interaction network and the following parameters:

● Search algorithm: ACO

● Search strategy: INES

● Node exceptions (K): 25

● Case exceptions (L): 25% of the samples, i.e. 39 in gene expression and 73 in methylation, respectively

**Figure 3:**
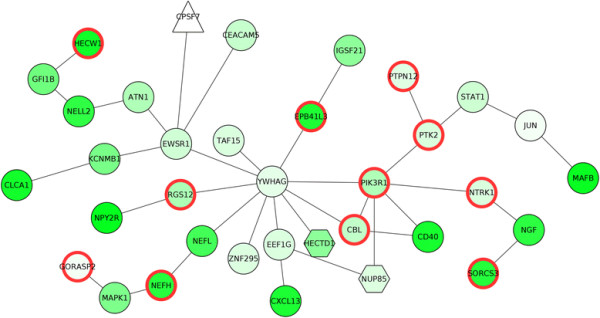
**Largest key pathway obtained for the colorectal cancer case study.** It consists of 35 proteins. The colorectal cancer associated genes are highlighted with red border. PI3K signaling associated genes are highlighted with blue border.

This resulted in a set of sub-networks (key pathways) enriched for genes exhibiting altered expression levels and altered DNA methylation. The biggest key pathway consisted of 35 genes, as depicted in Figure 3. To investigate whether the genes in this pathway had a significant role in colorectal cancer, we used the Ingenuity Pathway analysis (IPA) database (Ingenuity® Systems, www.ingenuity.com). We found it contains several colorectal cancer associated genes, which are highlighted in the network of Figure 3. Additionally, we identified several PI3K signaling associated genes in this key pathway (Table 1). PI3K signaling is over active in colorectal cancer and is a hotspot for therapeutic targets [14],[15].

**Table 1:** **PI3K signaling associated genes in the key pathway presented in Figure**3

**Gene**	**GO term**	**Source**
CBL	positive regulation of phosphatidylinositol 3-kinase signaling	Pubmed: 17003487
NGF	phosphatidylinositol-mediated signaling	Reactome
NTRK1	phosphatidylinositol-mediated signaling	Reactome
PIK3R1	phosphatidylinositol 3-kinase signaling	Pubmed: 7782332
PTK2	positive regulation of phosphatidylinositol 3-kinase signaling	Pubmed: 15166238

### Summary

The application case described here is meant to illustrate the applicability of KeyPathwayMiner 4.0 regarding its ability to identify sub-networks in Cytoscape, which are compact and possess many differentially active (expressed and methylated) genes, which are thus likely to be disease-specific. Note that this application example cannot be applied “blindly” to any kind of data set in a standard fashion. Data normalization and statistical pre-processing has to be taken into account for the individual omics data types and study designs. If given a set of indicator matrices and a network visualized with Cytoscape, KeyPathwayMiner combines the data and detects maximal aberrant sub-networks and visualizes them in Cytoscape.

## Discussion

With KeyPathwayMiner 4.0, we help the Cytoscape community to break out of the traditional way to analyze single omics studies together with interaction networks, by introducing a method that allows for a combined analysis of various types of omics studies. The new multi omics features were implemented outside the more computational intensive optimization steps of the KeyPathwayMiner algorithms, hence the runtimes reported [4],[8],[9] for previous versions of the software remain unaffected. We provided an example of how expression and methylation datasets could be combined in KeyPathwayMiner, however, we leave it up to the user to determine the best way to connect their different datasets. Quantifying the benefit of multi omics data integration in this context under different scenarios remains an open problem that we plan to tackle in the near future. Positive and negative custom node lists enable the utilization of prior expert knowledge. However, this artificially induced bias does not guarantee an actual modification of the output. Even though the *k* and *l* parameters have intuitive real world implications (noise in the network, noise in the omics data) it has sometimes been difficult to set these parameters to suitable values that fit a given data set. The introduced semi-automated result inspection method based on node coloring can track and visualize changes in pathway results according to different parameter values. The pre-processing, however, may be computationally intense, depending on user specifications. Finally, KeyPathwayMiner still works on discretized, indicator matrices. This leaves statistical pre-processing of the omics data to the user, as different omics data types require different normalizations and statistical analyses. For instance, RNA-seq gene expression data needs different statistical treatment than miRNA target enrichments or protein expression assays.

## Conclusion

Like previous versions, KeyPathwayMiner 4.0 solves the combinatorial optimization problem of extracting maximal deregulated graphs, but now with advanced features such as integration of multiple omics data sets, incorporation of prior expert knowledge, and improved visualization features.

## Availability and requirements

● **Project name:** KeyPathwayMiner 4.

● **Project home page:**http://keypathwayminer.mpi-inf.mpg.de, http://apps.cytoscape.org/apps/keypathwayminer.

● **Operating system(s):** Platform independent.

● **Programming language:** Java.

● **Other requirements:** Java 1.5 or higher, Cytoscape 2.*, 3.*

● **License:** GNU LGPL.

● **Any restriction to use by non-academics:** none.

### Ethics

No ethical issues declared. No experimental research on vertebrates or any regulated invertebrates. No research involving original human subjects, human material, or human data. We utilize publicly available data sets to demonstrate KeyPathwayMiner’s functionality.

## Competing interests

The authors declare that they have no competing interests.

## Authors’ contributions

NA, JP and JB designed the algorithms. NA implemented the algorithms. NA, JP and AJ developed the cytoscape app. AC, HD generated the in-house dataset. JP, EB, RB worked on the breast cancer dataset. RB worked on the colorectal cancer case study. All authors contributed to writing the manuscript. All authors read and approved the final manuscript.

## Authors’ information

Nicolas Alcaraz, Josch Pauling and Richa Batra are joint first authors.

Current affiliation: Center for non-coding RNA in Technology and Health, Section for Animal Genetics, Bioinformatics and Breeding, University of Copenhagen, Frederiksberg, Denmark. Alexander Junge's current affiliation: Center for non-coding RNA in Technology and Health, Section for Animal Genetics, Bioinformatics and Breeding, University of Copenhagen, Frederiksberg, Denmark.

## Additional files

## Supplementary Material

Additional file 1: Step-by-step instructions for KeyPathwayMiner 4.0. Click here for file

Additional file 2: Cytoscape network to be used with the step-by-step instructions. Click here for file

Additional file 3: Breast cancer expression files to be used with the step-by-step instructions. Click here for file
