# Supplementary material for: KeyPathwayMiner 4.0: condition-specific pathway analysis by combining multiple omics studies and networks with Cytoscape
Source: BMC Syst Biol. 2014 Aug 19;8:99. doi: 10.1186/s12918-014-0099-x (PMC4236746; doi:10.1186/s12918-014-0099-x)
Supplement: Additional file 1: — Step-by-step instructions for KeyPathwayMiner 4.0. [file s12918-014-0099-x-S1.docx]

**KeyPathwayMiner 4.0: Condition-specific pathway analysis by combining multiple omics studies and networks with Cytoscape**

Nicolas Alcaraz^1,4,5,#,*^, Josch Pauling^3,#^, Richa Batra^4,#^, Eudes Barbosa^4,7^, Alexander Junge^1,2,8^, Anne G.L. Christensen^5^, Vasco Azevedo^7^ , Henrik J. Ditzel^5,6^ and Jan Baumbach^1,2,4^

# Supplementary material

# STEP-BY-STEP INSTRUCTIONS

## Download and Install Cytoscape

- Cytoscape is available for download at <http://www.cytoscape.org/>.
- Installation instructions for Cytoscape are documented at http://cytoscape.org/documentation_users.html

## Install KeyPathwayMiner

- Open Cytoscape 3 and click on the App-tab in the main menu.
- Select AppManager and search for KeyPathwayMiner in search tab.
- Select KeyPathwayMiner 4.0 and click install.

## Network and Indicator dataset

We provide a sample network in additional_file_2.sif and a toy dataset (generated in-house) with additional file_[3-6].txt, for your convenience. The data set provided is differential protein expression and differential protein expression obtained by comparative, quantitative liquid chromatography tandem mass spectrometry (LC-MSMS)-based proteomics of human breast cancer stem cell lines.

## Upload network

- Click on File in the main Cytoscape menu bar.
- Select Import -> Network -> click on File-> browse to the network file Additional_file_2.sif”-> select the file -> click open.

## Upload datasets

- Once the KeyPathwayMiner plugin/app is installed, a tab will appear in the Cytoscape control panel named “KPM”.
- Click on the KPM-tab to upload datasets. One can click on the green plus sign to add more datasets.
- Do this and upload all the four datasets: Click on load-> browse for “additional_file_3.txt” -> click on green “plus”-button -> click on load -> browse for “additional_file_4.txt” -> click on ”plus” -> click on load -> browse for “additional_file_5.txt” -> click on ”plus”-> click on load -> browse for “additional_file_6.txt”

## Link the datasets

- To link the datasets click on the tab “Link” in the KPM menu bar.
- From the drop down menu of “Logically connect experiments”, choose the option “CUSTOM”. Now yellow nodes with the name of each of the dataset will appear in the left panel.
- You can left click in the empty space to add logical operators for setting up the combination formula. It will add blue nodes with default operator “OR”. One can change the type of operator (OR, AND, XOR) by left clicking on the node and selecting the option from the pop-up menu.
- Left click in empty space to add a logical operator. Click on the yellow node representing “additional_file_3” and drag the mouse to the blue node with logical operation “OR”. This will create an arrow between the two nodes. Now click on the yellow node representing “additional_file_4” and connect to the same “OR” node in a similar fashion.
- Repeat the above with “additional_file_5” and “additional_file_6”.
- Now join the two “OR” nodes with an “AND” operator. Right click on the empty space to add a blue node. Left click on this node and change the operator to “AND”.
- The formula should look as shown in Suppl. Fig. 1.

Supplementary Figure 1 - Combine formula to be used for the toy dataset


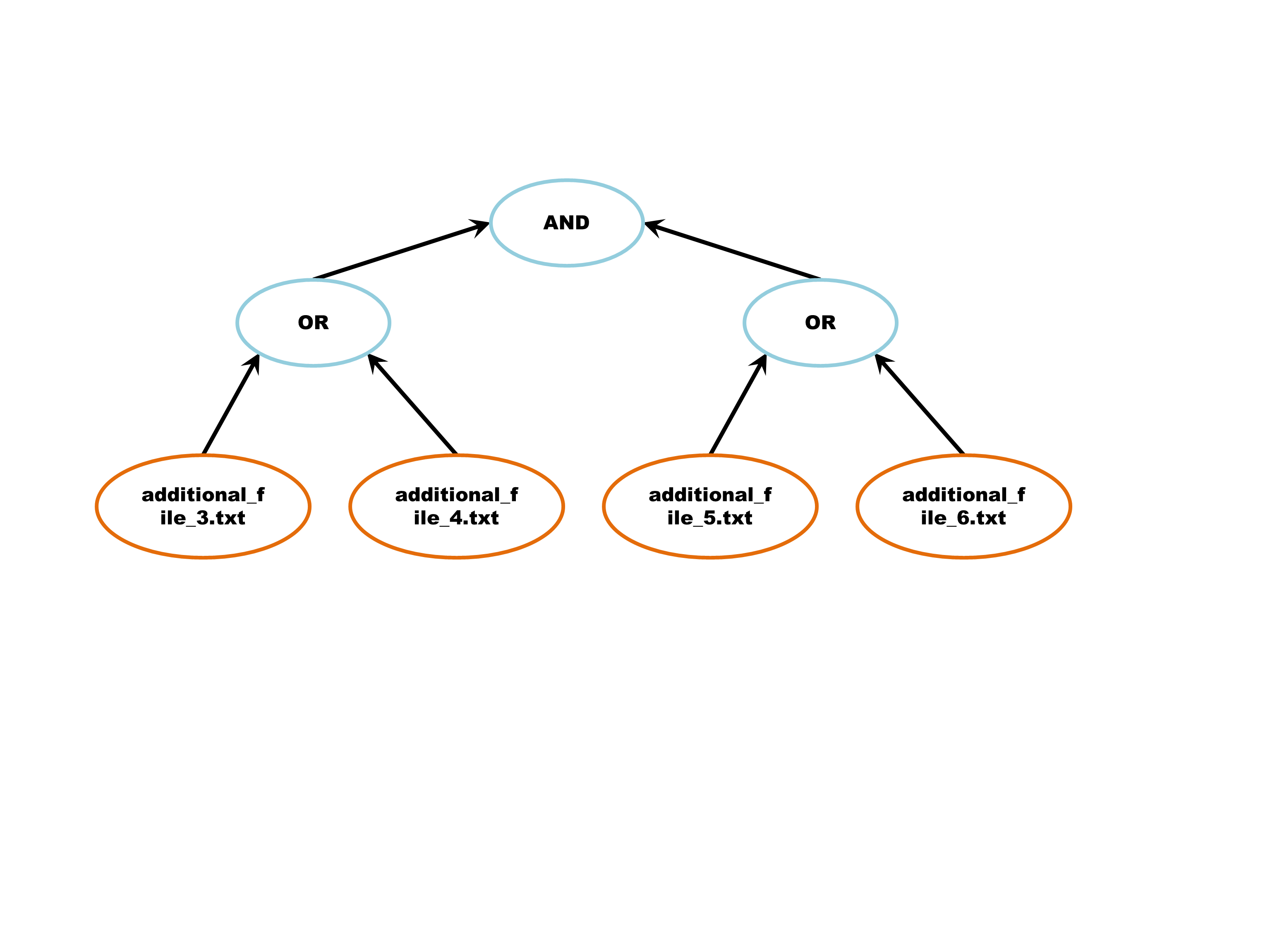


## Compute pathways

- Now click on the tab “Run” in the KPM menu bar.
- Keep the default parameters and click on “Search key pathways”
- A progress bar will appear.

## Results

- Once the pathway computation is complete a new tab “Results” will emerge in the KPM menu bar.
- Click on the biggest pathways that you see in the “Pathways table” and choose “view selected”.
- This pathway should look like the one below in Suppl. Fig. 2 (without the specific format).

A more detailed tutorial is available here: <http://keypathwayminer.mpi-inf.mpg.de/cyto3.html>


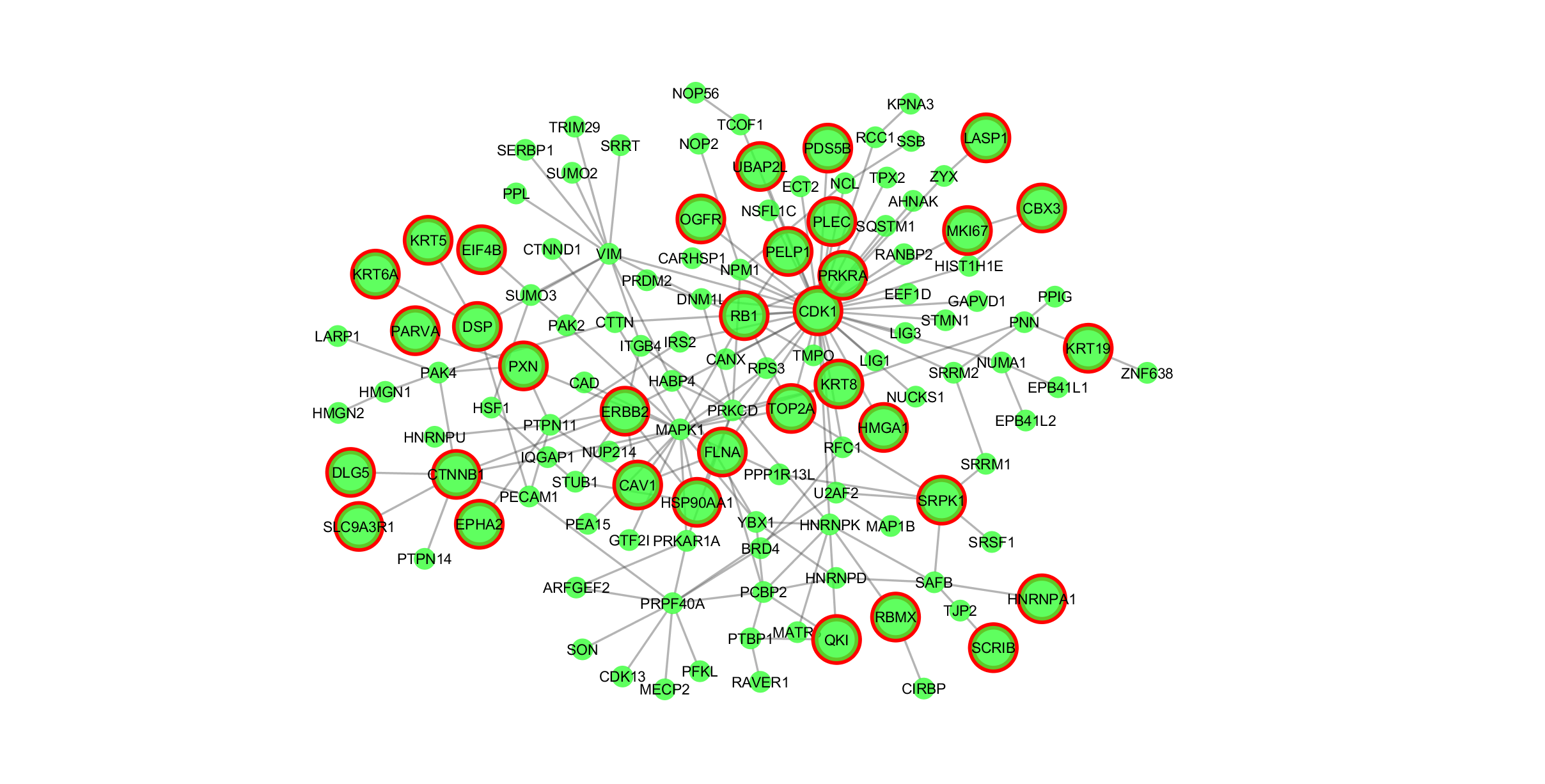


Supplementary Figure 2 - Resulting key pathway by using the toy dataset. Here, we highlight breast cancer associated genes using a red border.
